# State of Cardiac Implantable Electronic Devices in Congenital Heart Disease: A 2019 Update

**DOI:** 10.19102/icrm.2019.101207

**Published:** 2019-12-15

**Authors:** Johannes C. von Alvensleben, Kathryn K. Collins

**Affiliations:** ^1^Children’s Hospital Colorado; University of Colorado School of Medicine, Aurora, CO, USA

**Keywords:** Congenital heart disease, defibrillation, pacing, pediatrics

The prevalence of adult congenital heart disease (CHD) is directly attributable to the success of pediatric cardiology and congenital cardiac surgery in diagnosing and managing cardiac conditions in childhood. The observed success with this population has resulted in the application of electrophysiological techniques and therapies to an expanding population of patients, bypassing previous limitations secondary to size, underlying knowledge base, or both. Cardiac implantable electronic devices (CIEDs) are no exception and the last decade has seen a remarkable expansion into the CHD population. The 2014 Pediatric and Congenital Electrophysiology Society (PACES) and the Heart Rhythm Society (HRS) expert consensus statement on the recognition and management of arrhythmias in adult CHD^1^ and the 2018 American Heart Association/American College of Cardiology guidelines for the management of adults with CHD^2^ underscore that the care of these patients continues to become formalized. The year 2019 saw the release of several studies examining the impact of CIEDs in CHD with a focus placed on subcutaneous implantable cardioverter defibrillators (S-ICDs), epicardial devices, cardiac resynchronization therapy (CRT) systems, and leadless pacing.

## Subcutaneous implantable cardioverter defibrillators

The release of the S-ICD was heralded as a result of its utility in patients with limited vascular access and contraindications to the implantation of transvenous systems. This, the lack of intravascular components, and the concomitant need for lead extraction made the S-ICD particularly favorable in the CHD population, where patients are typically younger and frequently present with atypical venous return. This year saw the release of several larger series describing the acute and midterm outcomes of S-ICD implantation and served to enhance our understanding of this device type in this challenging group of patients.

At the 2019 HRS Scientific Sessions, a collaborative presentation organized together with the PACES described 116 pediatric and adult patients who had undergone S-ICD implantation, including 37 who had CHD.^3^ Among the entire cohort, the investigators found that 97.9% of patients experienced a successful first-shock cardioversion during implant testing and 92.5% demonstrated the delivery of appropriate therapy during a follow-up period of 33 months ± 29 months. Complications were comparable to those reported in other studies of CIEDs in this population, with 18.9% of patients experiencing any of a variety of selected complications. Notably, infections requiring device removal were low (1.7%) and no cases of endocarditis were reported. Female gender (hazard ratio: 2.76; 95% confidence interval: 1.22–6.21; p = 0.015) and a history of less than three previous implants (p = 0.039) were associated with the development of any complication but did not distinguish between the onset of infection or experience of inappropriate shock. These findings were similar to those described by Willy et al.^4^ in a single large-volume center review of 20 patients with congenital heart disease, emphasizing that implanter experience can reduce the occurrence of complications often found in this population.

Of note, a viable pacing option for bradycardia indications remains lacking in the S-ICD device profile, constituting a significant limitation for patients with CHD who frequently develop sinus node dysfunction earlier than in other comparably aged populations. Though not specifically involving the CHD population, 2019 saw the release of several additional studies examining the real-world combination of leadless pacemakers and S-ICDs for both the application of bradycardia pacing as well as the termination of ventricular tachycardia with antitachycardia pacing. Ito et al.,^5^ Ljunstrom et al.,^6^ and Baroni et al.^7^ all described the successful implantation and midterm follow-up outcomes of patients who received an S-ICD and the Micra™ leadless pacemaker (Medtronic, Minneapolis, MN, USA). The patient described by Ito et al. had prosthetic valve endocarditis precluding transvenous pacing but tolerated leadless pacing with no evidence of S-ICD crosstalk and displayed appropriate eligibility in all sensing vectors (ie, primary, secondary, and alternative) during ventricular pacing. Expanding upon this, Ljunstrom et al. demonstrated continued appropriate Micra™ (Medtronic; Minneapolis, MN, USA) behavior despite repeated appropriate S-ICD discharges for ventricular tachycardia. While these cases are encouraging, the lack of atrial leadless pacing continues to limit the overall utility of this technology setup in CHD. The release of a VDD pacing option is anticipated and will require further review.

Finally, Lüker et al.^8^ presented a remarkable collection of patients with dextrocardia and S-ICD implantation. With an estimated incidence of less than one in 10,000 live births, dextrocardia is a rare condition and, yet, 11 patients—including eight with CHD—were included in this research. The authors demonstrated that the use of an S-ICD was safe in this population, with no complications and one appropriate discharge for ventricular tachycardia.

## Epicardial devices

Epicardial pacing and defibrillation systems constitute a large percentage of devices placed in pediatric patients and those with CHD. While these options are required secondary to size and access limitations, the commonly cited complications of decreased lead longevity, elevated pacing thresholds, and the exclusion of future magnetic resonance imaging studies are well known. Cardiac strangulation, though reported, is considered a rare phenomenon.^9^ Mah et al.^10^ discussed a large series of patients from a single institution who all underwent coronary evaluation using either computed tomography (CT) scans or cardiac catheterization and discovered a higher incidence of coronary compression (5.5%) than that previously reported. The fact that the conduct of the study was initially prompted by the presentation of sudden death secondary to coronary compression underlies the seriousness of the issue. In total, 145 patients were examined and those included had a CT scan or coronary catheterization obtained for routine care or secondary to symptoms or chest X-ray (CXR) suggestive of coronary compression. Notable findings included the high specificity (96%) of a two-view CXR to identify “classic” patterns of leads with cardiac strangulation, described as a lead that courses leftward and posterior around the heart. This would be particularly relevant to ICD leads as they are frequently wrapped posteriorly in order to achieve appropriate defibrillation vectors. As such, these systems are considered to hold the highest risk of compression. No single modality (eg, CXR, CT, or catheterization) was perfect in identifying patients, having good specificity (96%, 93%, and 100%, respectively) but showing varying rates of sensitivity (57%, 100%, and 86%). As expected, catheter angiography had the highest positive predictive value (100%) in comparison with 44% for CXR.

Additional multicenter studies are required to further clarify the true prevalence of coronary compression and better identify the risks inherent with pacing-only versus defibrillation systems. There remain many unanswered questions in terms of which population(s) should be studied; which test(s) should be utilized; how frequently one should obtain these tests; and, finally, whether the removal of the epicardial system corrects the coronary compression or not.

## Cardiac resynchronization therapy

CRT has long been an accepted adjunct modality in adult patients with left ventricular (LV) dysfunction, particularly in those with left bundle branch block. Its utility in CHD has been more problematic, however, owing both to the heterogeneity of eligible patients as well as a lack of accepted guidelines. Beginning with the 2014 PACES/HRS expert consensus statement on arrhythmias in adult CHD,^1^ more formalized recommendations have emerged and set the stage for increased use of this technique. In particular, the previously published BLOCK-HF trial^11^ resulted in a confirmation that CRT is reasonable in patients with LV ejection fractions of less than 50% and pacing burdens of more than 40% after heart failure medications had been trialed, opening the door for CRT to be deployed in a large CHD population. Additionally, specific recommendations for patients with single-ventricle and systemic right-ventricle (RV) presentations were included.

In 2019, a number of notable articles on the long-term outcomes of CRT in adult CHD,^12^ the treatment of subpulmonary RV dysfunction using CRT,^13^ minimally invasive approaches to CRT deployment in single RVs,^14^ and a novel morphology-based strategy for evaluating CRT outcomes^15^ were released.

In a single-center observational study comparing adult CHD patients to adults with ischemic or nonischemic cardiomyopathy undergoing CRT, Leyva et al. reported no significant difference was observed in the areas of total mortality, cardiac mortality, and heart failure hospitalization after CRT.^12^ The total number of ACHD patients was small (n = 23) and there was a lower number of left bundle branch block patients in this group than in the comparison group. However, the majority of participants had moderate to complex CHD, with a significant number of systemic RV patients (n = 9; 39.1%) as well as two single-ventricle patients (8.7%) and three patients (13%) with epicardial systems. More than half of all included adult CHD patients were upgraded from pacemakers to CRT systems.

A special report published by Janoušek et al.^13^ detailed permanent RV CRT in a series of six symptomatic patients with chronic RV dysfunction and CHD. The mechanism of LV electromechanical dyssynchrony causing dysfunction and impaired remodeling is the basis for traditional CRT. This report outlined the technique of RV resynchronization by examining patients with right bundle branch block and intact atrioventricular conduction. Latest RV activation during atrial pacing was sought and atrioventricular delays were set to achieve maximal fusion with intrinsic ventricular activation and the narrowest QRS duration. During a median follow-up period of 14.3 months, both acute and chronic responses were observed. In particular, improved RV dP/dt_max_, longer RV filling time, and a lower RV index of myocardial performance were observed. The authors correctly proposed that prospective trials will be necessary to better elucidate long-term outcomes.

An optimal strategy for CRT in patients with systemic RVs has not been identified. The 2014 consensus document^1^ awards a class IIa recommendation in the setting of an RV ejection fraction of 35% or less with accompanying right bundle branch block and a QRS duration of 150 ms or more. Class IIb recommendations extend to patients with higher ejection fractions but a prolonged QRS duration or greater than 40% ventricular pacing. In order to better identify optimal pacing sites, Moore et al.^13^ presented a retrospective review of six patients undergoing a hybrid transcatheter–surgical approach to assess for latest RV activation in the setting of systemic RV failure. Detailed endocardial electroanatomical mapping of the systemic RV was followed by focused epicardial mapping. The exact site of latest endocardial activation was variable but localized to the basolateral RV in all cases. Sites of latest activation tended to be more superior during contralateral ventricular pacing in comparison with intact atrioventricular conduction (p = 0.06). QRS durations were reduced by a median of 23.8% and, notably, 66% of patients initially referred for transplant evaluation no longer met the criteria following CRT. While this technique revealed that more precise mapping can be achieved in this select population, it required epicardial access and the results of the long-term comparison to traditional techniques are unknown.

Finally, Miyazaki et al.^15^ presented a retrospective review of 24 patients in whom they tested their previously described “morphology-based” strategy for the placement of CRT leads in CHD. According to the authors, the leads should be placed laterally on opposite sides of both ventricles in patients with short-axis dyssynchrony and single-ventricular physiology, whereas they should be placed at the farthest sites along the longitudinal direction in patients with long-axis dyssynchrony of the RV. The technique of lateral separation in single-ventricle patients has been previously described by Cecchin et al.,^16^ with the use of both sternotomy and lateral thoracotomy frequently required to obtain an optimal lead position. In their study, Miyazaki et al. describe the achievement of an impressive 63% responder rate, though the nonoptimal lead position was common and a leading cause of nonresponse.

## Leadless pacing

Leadless transcatheter pacing systems have been studied in adults with structurally normal hearts in both Europe and the United States. These systems have demonstrated low complication rates at both the time of implantation and throughout the short- to midterm follow-up. However, there is limited published research on the use of this technology in those with CHD. This year saw the release of an additional two papers examining this topic, including in the first pediatric patient^17^ and a small series of three adults with CHD.^18^ Both papers describe the use of the Micra™ transcatheter pacing system (Medtronic, Minneapolis, MN, USA).

McCanta et al. introduced a 12-year-old, 37-kg girl with a history of repaired tetralogy of Fallot with pulmonary atresia and postoperative high-grade heart block status following initial epicardial pacemaker placement at five months. When she eventually developed ventricular lead fracture, the patient had undergone four previous sternotomies and had an intermittent—though persistent—need for pacing. Procedurally, device repositioning was required a total of seven times before acceptable parameters could be achieved (1.25 V at 0.24 ms, sensed R-wave of 4.4 mV, and pacing impedance of 480 Ω). However, pacing thresholds continued to rise for two weeks postprocedure before eventually plateauing, decreasing, and stabilizing at 2.0 V at 0.24 ms at 12 weeks postimplantation.

The adult CHD literature is only slightly more robust with case reports and small series from which to draw insight. Moore et al.^18^ published a series of three adult patients with intracardiac shunts or tricuspid valve disorders who underwent leadless pacemaker placement. Pacing indications included sinus node dysfunction in two and permanent atrial fibrillation with atrioventricular block in one. Initially rising lead thresholds were noted in this series as well, though they eventually became acceptable and no complications were reported during follow-up.

Careful patient selection is paramount as, in its current state, leadless pacing is only available in the ventricle. Prolonged nonsynchronous pacing has been repeatedly demonstrated to be inferior to synchronous pacing in CHD. Additionally, questions about device extraction versus redundant implantation over a long lifetime remain.

## Summary

This year has brought about several advancements in our knowledge of CIEDs in CHD along with introductions to technologies and techniques that will require further refinement. The S-ICD has been shown to be a safe and effective alternative to transvenous or epicardial systems and the addition of leadless pacemakers may make this system even more broadly applicable. Coronary compression secondary to epicardial pacing or defibrillation systems is more common than previously suspected but noninvasive evaluation with CXR provides some predictive power prior to cardiac CT or angiography. CRT continues to be difficult to study in the CHD population but techniques have emerged to expand the indications and therapeutic potential for this well-described concept. Leadless pacing is also growing as an area of interest but currently remains an uncommon therapy in patients with CHD.
